# Occurrence and outcome of COVID-19 in AIRD patients on concomitant treatment with Tofacitinib- results from KRA COVID COHORT (KRACC) subset

**DOI:** 10.1186/s41927-023-00345-8

**Published:** 2023-07-26

**Authors:** Pramod Chebbi, Vineeta Shobha, Vijay K Rao, Vikram Haridas, Ramya Janardana, Benzeeta Pinto, Sharath Kumar, Abhishek Patil, Roopa Tekkatte, Manasa Salanke, K M Mahendranath

**Affiliations:** 1 Department of Rheumatology, SDMCMSH, SDM University Dharwad, Dharwad, India; 2 Department of Clinical Immunology and Rheumatology, St.John’s Medical college Hospital, Sarjapur Road, Bengaluru, 560034 India; 3 Department of Rheumatology, Manipal Hospital, Bengaluru, India; 4 Arthritis Superspeciality Center, Hubli, India; 5 OPTIMA Rheumatology & Arthritis clinic, Bengaluru, India; 6 Aster RV hospital, Bengaluru, India; 7 ChanRe Rheumatology and Immunology Centre, Bangalore, India; 8 Samarpan Health center, Bangalore, India

**Keywords:** Autoimmune rheumatic diseases, SARS-CoV-2 infection, Covid-19, Concomitant tofacitinib treatment

## Abstract

**Introduction:**

We assessed the risk factors and outcome of COVID-19 in patients with autoimmune rheumatic diseases(AIRD) who contracted infection while on background treatment with tofacitinib.

**Methods:**

This is a non-interventional, cross-sectional, questionnaire based telephonic study which included consecutive AIRD patients on tofacitinib co-treatment. Data related to the AIRD subset, disease modifying anti rheumatic drugs(DMARDs) including glucocorticoids and comorbidities, was collected from 7 rheumatology centers across Karnataka during the second wave of COVID-19 pandemic. The information about COVID-19 occurrence and COVID-19 vaccination was recorded.

**Results:**

During the study period (Jun-July 2021), 335 AIRD patients (80.6% female) on treatment with tofacitinib were included. The mean duration of tofacitinib use was 3.4+/-3.1months. Thirty-six(10.75%) patients developed COVID-19. Diabetes mellitus (p = 0.04 (OR 2.60 (1.13–5.99)) was identified as a risk factor for COVID-19 in our cohort. Almost half of our cohort was COVID-19 vaccinated with at least one dose, with resultant decline in incidence of COVID-19(OR 0.15 (0.06–0.39) among the vaccinated. Recovery amongst COVID-19 infection group was 91.2%.

**Conclusions:**

The subset of AIRD patients who were on treatment with tofacitinib were found to have a higher rate of COVID-19 infection as compared to our KRACC cohort. Pre-existing comorbidity of diabetes mellitus was the significant risk factor in our cohort. This subset of the KRACC cohort shows RA patients had a lesser infection and PsA patients had a higher infection.

**Supplementary Information:**

The online version contains supplementary material available at 10.1186/s41927-023-00345-8.

## Key messages

1. Higher prevalence of COVID-19 was noted amongst AIRD patients on concomitant therapy with tofacitinib as compared to the general population during the same time period in the same geographic region.

2. Diabetes mellitus was identified as a significant risk factor for COVID-19.

3. COVID-19 vaccination protects AIRD patients on treatment with tofacitinib.

**Supplementary Information:**

The online version contains supplementary material available at 10.1186/s41927-023-00345-8.

## **Introduction**

The ongoing COVID-19 pandemic has posed an immense burden on the healthcare systems across the globe. As we write this report, the infection caused by SARS-CoV2 has become akin to an endemic infection in several parts of the world [1]. Dysfunctional immune response has been one of the key factors in the pathogenesis, progression and complications of the disease process. It is now clearly evident that controlling the aberrant inflammatory response is essential to reduce morbidity and mortality in COVID-19 [2]. Therefore, various immunomodulatory medications such as glucocorticoids(GC), hydroxychloroquine (HCQ), biologics such as tumor necrotic factor inhibitors (TNFi), tocilizumab and Janus kinase inhibitors (JAKinib) have been variably employed and experimented in its management strategies [3].[4] Indeed, baricitinib, a JAKinib, has Food and Drug Administration(FDA) emergency use approval for treatment of moderate-severe COVID-19 [5]. Another JAKinib, tofacitinib, demonstrated reduction in all-cause mortality and respiratory failure at 4 weeks for the treatment of COVID-19 pneumonia through STOP-COVID, a randomized placebo controlled trial [6].

Tofacitinib is a prototype JAK inhibitor that predominantly inhibits Janus kinase 1 & 3 (JAK1 and JAK3) and to a lesser extent JAK 2 [7]. Currently, tofacitinib is approved for the treatment of rheumatoid arthritis, psoriatic arthritis, juvenile idiopathic arthritis, ulcerative colitis and ankylosing spondylitis. The generic tofacitinib is extensively prescribed in our country for treatment of these rheumatological disorders.

The influence of concomitant immunosuppressant therapy on COVID-19 has been reported through large registries, cohort studies and databases [8, 9]. Largely, except for few, they do not seem to impact the occurrence as well as outcome of COVID-19. The impact of COVID-19 on autoimmune rheumatic diseases(AIRD) patients while on current treatment with tofacitinib has not been specifically looked into. Here, we report incidence, risk factors and outcome of COVID-19 in this subset of AIRD patients[10].

## Methods

Karnataka, a southern state in India, is conducting a multicentre prospective longitudinal study to ascertain risk factors associated with COVID-19 in AIRD patients viz. Karnataka Rheumatology Association COVID Cohort (KRACC) [11, 12].

### Study design and participant population

This is a non-interventional, cross-sectional, questionnaire-based telephonic substudy of KRACC involving 7 specialist rheumatology centers. Consecutive adult AIRD patients who were carrying current prescriptions of tofacitinib for any indication were invited. All others not on concomitant treatment with tofacitinib and non-autoimmune rheumatologic disorders were excluded. This study was conducted during the second COVID-19 wave, which had a higher mortality in India (June-July 2021).

### Data Collection

Data were recorded using a structured case record form modified for this study (Supp. Figure 1). Apart from Information pertaining to tofacitinib, details regarding the AIRD subset, the current and or past use of immunosuppressive medications were recorded. All comorbidities, including pre-existing lung diseases were noted. Other medications including the use of antihypertensives such as angiotensin-converting enzyme inhibitor (ACEi) or angiotensin receptor blockers (ARBs) and antiplatelet agents were recorded. The diagnosis of COVID-19 was as per Reverse Transcription-Polymerase Chain Reaction (RT-PCR) or Rapid Antigen Test(RAT). COVID-19 testing protocols for symptomatic infection or exposed contacts and vaccination were as per Government of India recommendations. Outcome of COVID-19 and COVID-19 vaccination information was recorded.

### Ethics approval

The current analysis is part of a study titled ‘Role of HCQ in COVID-19 pandemic in Rheumatologic disorders study”. Respective Institute’s ethics committee (Sri DharmasthalaManjunatheshwara College of Medical Science and Hospital − 63/2020, St. John’s Medical College, Bangalore - IERB No-127/2020, Chanre Rheumatology and Immunology Center& Research - CRICR/SN-130/099/2020, Manipal Hospitals − 07/2020) approved this study and waiver of consent was obtained.

### Statistics

Descriptive statistics were reported as mean and standard deviation for continuous variables, number, and percentages for categorical variables. Association of COVID-19 positive with clinical characteristics of the study population was assessed using Chi-square/Fisher’s exact test as appropriate. Student’s t-test was used to compare the means between COVID-19 and non-COVID-19 groups. OLS (Ordinary Least Squares) regression analysis was performed, and Odds Ratio(OI) along with 95% confidence interval (CI) was reported. P value < 0.05 was considered significant. Statistical analyses were carried out using SPSS version 25.0 (IBM headquartered in Armonk, New York).

## **Results**

### Cohort characteristics

A total of 335 patients (80.6% female, mean age 48.6 ± 12.1 years) on current treatment with tofacitinib for various rheumatological disorders from 7 participating specialist rheumatology centers were included. Around 3/4th of patients were rheumatoid arthritis(RA), other disease subsets are represented in Table 1. The mean duration of AIRD was 77.1 ± 72.9 months and that of tofacitinib use was 3.39 ± 3.11months. In addition to tofacitinib, the majority of patients were on additional therapy, methotrexate being the most frequent(68.96%)(supp. Figure 2, supp.Table 1). Current and prior GC therapy was noted in 150/335(44.7%), of which 13/36 (36.1%) were infected group.

**Table 1:** Descriptive data and outcome of COVID-19

Descriptive Statistics N = 335(%)		
**Age in years (mean** **±** **SD)**		48.62 ± 12.07
**Gender M(F)**		65 (270)
**Duration of AIRD in months**	**Less than 24 Months**	60 (17.91%)
	**Greater than or Equal to 24 Months**	275 (82.09%)
**Smoking History**		5 (1.49%)
**DM**		43 (12.84%)
**HTN**		71 (21.19%)
**Obesity BMI > 30**		11 (3.28%)
**Asthma/ILD**		9 (2.69%)
**Diagnosis**	**RA**	246 (73.43%)
	**PsA**	29 (8.66%)
	**SpA**	35 (10.45%)
	**Others**	25 (7.46%)
**COVID-19 Infected Patients** **(N = 36)**		
**Hospitalization**		10 (27.78%)
**O2 therapy**		7 (19.44%)
**ICU admission**		3 (8.33%)
**Mortality (Death)**		3 (8.33%)

AIRD: Autoimmune Rheumatic Diseases; DM: Diabetes Mellitus, HTN: Hypertension, BMI- Body Mass Index, ILD: Interstitial lung disease, RA: Rheumatoid Arthritis; PsA: Psoriatic Arthritis; SpA- Spondyloarthritis; ICU: Intensive care unit

### Infection

Overall, the prevalence of COVID-19 in this cohort was 36/335(10.7%), RA had a lower incidence (221(73.9%), p < 0.01) as against patients with psoriatic arthritis(PsA) (11.1%, p < 0.01). Comparison of COVID-19 and non-infection group is represented in Table 2. Concomitant therapy with GC and HCQ did not influence COVID-19 occurrence, however those on concomitant methotrexate prescription had a lower incidence(p = 0.04; OR 0.46 (0.23–0.92))(Supp Table 2). Statistically significant variables were used for the infection model as independent variables and infection outcome as a dependent variable. Diabetes mellitus (p = 0.04, (OR 2.60 (1.13–5.99)) was identified as risk factors for developing COVID-19 in our cohort. Recovery among the infection group was 91.6%. Three patients succumbed to COVID-19 in our cohort. Among these three patients, two had underlying comorbidities of hypertension and obesity, one had diabetes mellitus as well. Third patient didn’t have any underlying comorbidity or additional risk factors. There were no thromboembolic events in our infection group.

**Table 2:** Comparison of COVID19 and non-infection groups among AIRD patients on background therapy with tofacitinib

Variables N = 335	COVID19 N = 36	Non-COVID19 N = 299	p-value	Odds Ratio
**Age in years**	51.5 ± 12.73	48.28 ± 11.96	NS^#^	
**Gender M (F)**	10 (26)	55 (244)	NS^*^	1.71 (0.78–3.74)
**Duration of AIRD in months**				
**< 24 Months**	5 (13.89%)	55 (18.39%)	0.34^$^	0.72 (0.27–1.92)
**≥ 24 Months**	31 (86.11%)	244 (81.61%)		
**Smoking**	0	5 (1.67%)	NS^$^	-
**DM**	9 (25.0%)	34 (11.37%)	0.04^*^	2.60 (1.13–5.99)
**HTN**	11 (30.56%**)**	60 (20.07%)	NS^*^	1.75 (0.82–3.76)
**Obesity BMI > 30**	3 (8.33%)	8 (2.68%)	NS^$^	3.34 (0.84–13.08)
**Asthma/ILD**	2 (5.56%)	7 (2.34%)	NS^$^	4.22 (1.38–12.94)
**Diagnosis**				
**RA**	25 (69.44%)	221 (73.91%)	< 0.0001*	0.8 (0.38–1.71)
**PsA**	4 (11.11%)	25 (8.36%)	< 0.0001$	1.37 (0.45–4.19)
**SpA**	6 (16.67%)	29 (9.7%)	NS^*^	1.86 (0.72–4.85)
**Others**	1 (2.78%)	24 (8.03%)	NS^$^	0.33 (0.04–2.50)
**Hospitalization**	10 (27.78%)	0	NA	-
**Medications**				
**ACEi**	0	2 (0.67%)	NS^$^	-
**ARB**	5 (13.89%)	14 (4.68%)	NS^$^	3.28 (1.11–9.73)
**Glucocorticoid**	13 (36.11%)	137 (45.82%)	NS^*^	0.67 (0.33–1.37)
**Methotrexate**	19 (52.78%)	212 (70.9%)	0.04^*^	0.46 (0.23–0.92)
**HCQ**	7 (19.44%)	105 (35.12%)	NS^*^	-
**Leflunomide**	5 (13.89%)	43 (14.38%)	NS^$^	0.96 (0.35–2.61)
**Apremilast**	0	2 (0.67%)	NS^$^	-
**Iguratimod**	1 (2.78%)	1 (0.33%)	NS^$^	8.51 (0.52-139.61)
**Sulfasalazine**	3 (8.33%)	9 (3.01%)	NS^$^	2.93 (0.76–11.36)
**Aspirin**	1 (2.78%)	1 (0.33%)	NS^$^	8.51 (0.52-139.61)
**Anticoagulants**	0	1 (0.33%)	NS^$^	-
**Other Drugs**	18 (50.0%)	157 (52.51%)	NS^*^	0.9 (0.45–1.81)
**Vaccination status**				
**COVID-19 vaccination**	5 (13.89%)	156 (52.17%)	< 0.0001^*^	0.15 (0.06–0.39)

The above is the comparison of parameters between patients who were Infected & Non Infected. To find the Statistical difference ^#^t-test (http://vassarstats.net/tu.html) is used for Continuous variables & ^*^Chi-square test (http://vassarstats.net/newcs.html) & ^$^fisher exact (http://vassarstats.net/tab2x2.html) for categorical variables

DM: Diabetes Mellitus; HTN- Hypertension; BMI- Body Mass Index; ILD-Interstitial Lung Disease; RA: Rheumatoid Arthritis; PsA: Psoriatic Arthritis; SpA- Spondyloarthritis; ACEi -Angiotensin-converting enzyme inhibitor; ARBs-Angiotensin receptor blockers; HCQ- Hydroxychloroquine

### Vaccination

Almost half of our cohort was COVID-19 vaccinated with at least one dose at the time of participation in this study. There was significantly reduced incidence of COVID-19 amongst the vaccinated AIRD patients [p < 0.0001,(0.15 (0.06–0.39)] (Table 2). Mean time gap between vaccination and COVID-19 was 72.8 ± 64.93 days. All three patients with mortality had not received COVID-19 vaccination.

## Discussion

This prospective study in AIRD patients on concomitant tofacitinib therapy was envisaged when sporadic reports of beneficial effects of JAKinibs emerged for the treatment of COVID-19 pneumonia. From our ongoing KRACC study, we preferentially recruited patients who were on tofacitinib prescription for any indication. We found prevalence of infection (10.75%), which appears higher than the general population (4.3%) and among AIRD patients during the first COVID-19 wave (5.7%) but we couldn’t compare with identical cohorts [11]. Wang et al., in a systematic meta-analysis which included data from 26 studies and about 2000 patients, reported 1.5 times higher risk for COVID-19 in rheumatic patients (OR = 1.53, 95% CI 1.24–1.88) [13]. We could not find similar publications evaluating the impact of background tofacitinib on incidence of COVID-19 in AIRD patients.

Real-world data from the COVID-19 Global Rheumatology Alliance registry had a very small number of patients on JAKinibs and reported them alongside bDMARDs. They report a decreased risk of hospitalization in AIRD patients on JAKinibs, either as monotherapy or in combination with csDMARDstreatment [3].

High risk of infections among patients receiving high doses of GC and intense immunosuppressants like cyclophosphamide and biologicals has been well-established in many studies [14–16]. In our cohort, none of the IS therapiesinfluenced either the incidence or the outcome of COVID-19. However, methotrexate as a concomitant csDMARD was associated with a lesser risk of COVID-19 in our study population similar to a few case series and observations [17, 18]. Methotrexate usage during COVID-19 pandemic has a favourable outcome possibly because of downregulating the receptor for angiotensin-converting enzyme (ACE)-2 and reduced severe inflammatory reaction [17, 18].

The international registry of IBD (SECURE IBD) diseases reported similar incidence of hospitalization due to COVID-19 among IBD patients treated with or without tofacitinib (21.6% vs. 23.3%) [19]. Meta-analysis of severe COVID-19 infection among IBD patients who were using tofacitinib revealed RR 0.81 (95% CI 0.49 to 1.33, p = 0.40) indicating no significant association between their use and severe outcomes [20]. In our cohort hospitalization was required in 10/36 (30.5%), however this should be interpreted with caution, as AIRD patients on IS therapy may be apprehensive due to immunomodulated status and preferentially seek hospitalization. On the other hand, during our study time period, there was an acute shortage of hospital beds, oxygen supply, and intensive care facilities in our country.

A recent meta-analysis involving 42 studies and 423,117 patients has confirmed the influence of various comorbidities including diabetes as a significant risk factor for COVID-19 mortality. We found diabetes mellitus (OR2.60 (1.13–5.99)) as an independent risk factor for infection in our cohort [21].

Further, the risk of thromboembolic events is of particular concern in patients with severe COVID-19, and venous thromboembolism has been identified as a safety risk for tofacitinib. We did not observe any events of thromboembolism in our cohort.

Three patients in our cohort died (3/36(8.3%). This is much higher than the mortality in the general population in the same region. We postulate that this could be attributable to the cumulative burden of IS rather than tofacitinib alone.

In our cohort, single-dose vaccination had significant protection against COVID-19 infection(p < 0.0001). The second dose of vaccination was just completed or was due for the majority of patients and hence its efficacy couldn’t be extrapolated.

The strengths of our study include prospective collection of data, in a well-defined cohort, a large sample size of more than 300 tofacitinib prescribed AIRD patients from specialist rheumatology centres, and the inclusion of only RT-PCR/ RAT confirmed cases for the definition of infection. Further, we were able to study the impact of vaccination in our cohort. Limitations include reporting bias as immunosuppressed patients are more likely to test for COVID-19 and seek hospitalisation. We have not evaluated the impact of socio-demographic factors and personal protective measures which may likely have influenced the incidence of COVID-19. In our study only 50% were vaccinated and all 3 who died were unvaccinated. We could compared only few PsA patients with a higher number of RA patients, hence need a larger study for complete inference.

## Conclusions

The subset of AIRD patients who were on treatment with tofacitinib was found to have a higher rate of COVID-19 infection as compared to our KRACC cohort. Pre-existing comorbidity of diabetes mellitus was the significant risk factor in our cohort. This subset of the KRACC cohort shows RA patients had a lesser infection and PsA patients had a higher infection.

## Electronic supplementary material

Below is the link to the electronic supplementary material.

Supplementary Material 1: Case Record Form

## Data Availability

The authors confirm that the data supporting the findings of this study are available within the article (and/or) its supplementary materials. The datasets generated and analysed during the current study are available from the corresponding author on reasonable request. The data and materials are available to all authors.

## Abbreviations

COVID 19: CoronaVirus Disease 2019
AIRD: Autoimmune Rheumatic Diseases
KRACC: Karnataka Rheumatology Arthritis COVID Cohort
DMARDs: Disease Modifying Anti Rheumatic Drugs
SARS-CoV2: Severe Acute Respiratory Syndrome CoronaVirus 2
GC: Glucocorticoids
HCQ: Hydroxychloroquine
TNFi: Tumor Necrotic Factor inhibitor
JAKinib: Janus kinase inhibitors
FDA: Food and Drug Administration
ACEi: Angiotensin-Converting Enzyme inhibitor
ARBs: Angiotensin Receptor Blockers
RT-PCR: Reverse Transcription-Polymerase Chain Reaction
RAT: Rapid Antigen Test
OLS: Ordinary Least Squares
OI: Odds Ratio
CI: Confidence interval
