# Supplementary material for: Occurrence and outcome of COVID-19 in AIRD patients on concomitant treatment with Tofacitinib- results from KRA COVID COHORT (KRACC) subset
Source: BMC Rheumatol. 2023 Jul 26;7:22. doi: 10.1186/s41927-023-00345-8 (PMC10369741; doi:10.1186/s41927-023-00345-8)
Supplement: Supplementary file 1 — Supplementary Material 1: Case Record Form [file 41927_2023_345_MOESM1_ESM.docx]

**Supplementary Figure 1:** Case Record Form

**Supplementary Figure-2:** Horizontal Bar graph showing concomitant non-tofacitinib csDMARDs distribution in RA, PsA & SpA patients using plotted excel.

**
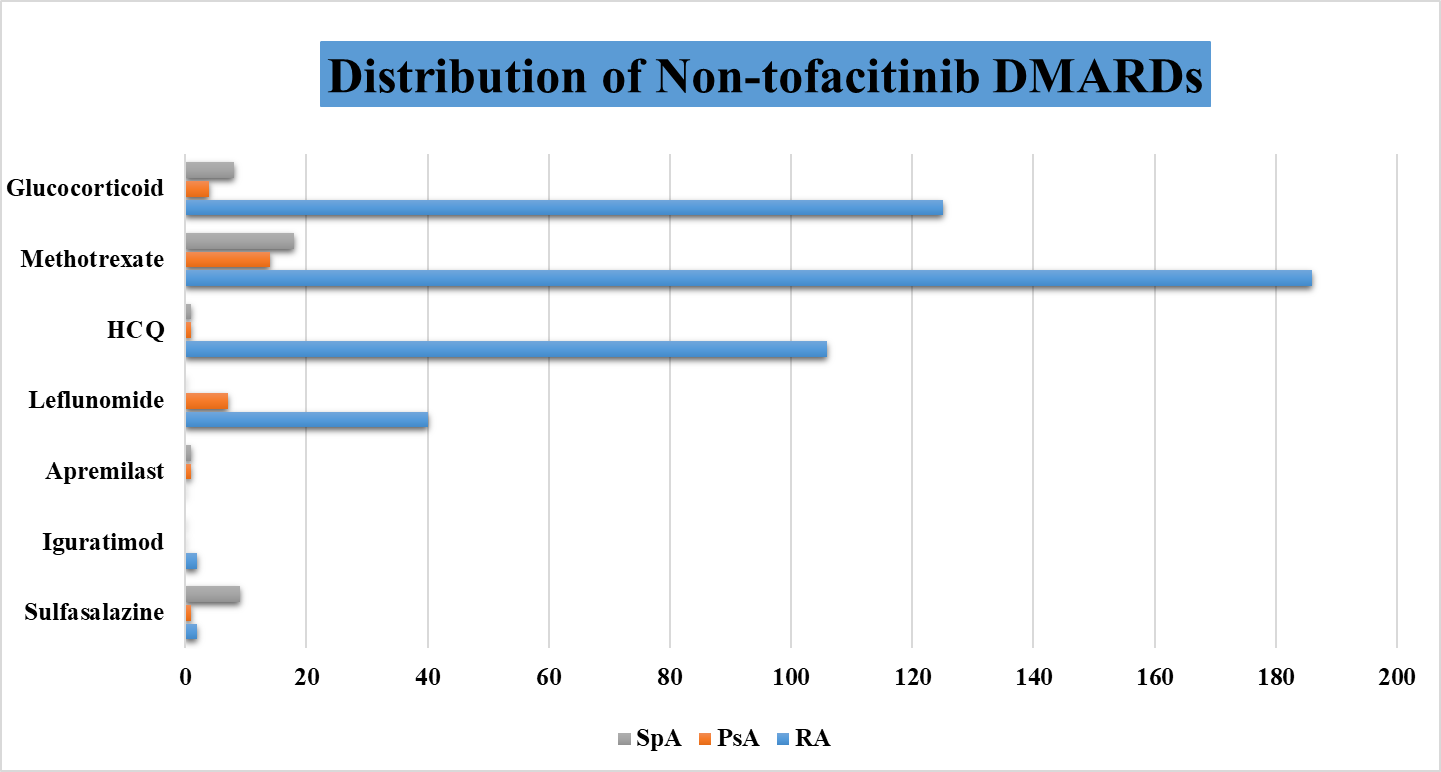
**

Abbreviations- RA: Rheumatoid Arthritis; PsA: Psoriatic Arthritis; SpA- Spondyloarthritis, HCQ: Hydroxychloroquine

**Supplementary Table 1: Disease-wise distribution of concomitant non-tofacitinib DMARDs**

|  | **RA N=246** | **PSA N=29** | **SPA N=35** |
| --- | --- | --- | --- |
| **Glucocorticoids** | 125 (50.81%) | 4 (13.79%) | 8 (22.86%) |
| **Methotrexate** | 186 (75.61%) | 14 (48.28%) | 18 (51.43%) |
| **Leflunomide** | 40 (16.26%) | 7 (24.14%) | 0 |
| **Apremilast** | 0 | 1 (3.45%) | 1 (2.86%) |
| **Iguratimod** | 2 (0.81%) | 0 | 0 |
| **SSZ** | 2 (0.81%) | 1 (3.45%) | 9 (25.71%) |
| **HCQ** | 106 (43.09%) | 1 (3.45%) | 1 (2.86%) |

**Abbreviations:** RA: Rheumatoid Arthritis; PsA: Psoriatic Arthritis; SpA- Spondyloarthritis; HCQ- Hydroxychloroquine; SSZ- Sulfasalazine

**Supplementary Table 2: Regression Analysis with respect to COVID Infection.**

| **Dep. Variable:** | Outcome | **R-squared:** | 0.029 |
| --- | --- | --- | --- |
| **Model:** | OLS | **Adj. R-squared:** | 0.017 |
| **Method:** | Least Squares | **F-statistic:** | 2.446 |
| **No. Observations:** | 335 | **Prob (F-statistic):** | 0.0463 |
| **Df Residuals:** | 330 | **Log-Likelihood:** | -77.78 |
| **Df Model:** | 4 | **AIC:** | 165.6 |
| **Covariance Type:** | nonrobust | **BIC:** | 184.6 |

|  | coef | **std err** | **t** | **P>\|t\|** | **[0.025** | **0.975]** |
| --- | --- | --- | --- | --- | --- | --- |
| **Intercept** | 0.1509 | 0.044 | 3.408 | 0.001 | 0.064 | 0.238 |
| **C(DM) [T.1]** | 0.1106 | 0.051 | 2.17 | 0.031 | 0.01 | 0.211 |
| **C(RA) [T.1]** | -0.0095 | 0.046 | -0.208 | 0.835 | -0.099 | 0.08 |
| **C(PSA) [T.1]** | -0.0004 | 0.07 | -0.006 | 0.995 | -0.138 | 0.137 |
| **C(Methotrexate) [T.1]** | -0.0734 | 0.038 | -1.956 | 0.051 | -0.147 | 0 |

**Estimated Model:** ŷ = 0.1509+ (0.1106) * C(DM) [T.1] + (-0.0095) * C(RA) [T.1] + (-0.0004) * C(PSA) [T.1] + (-0.0734) * C(Methotrexate) [T.1]

The above regression result was obtained by running the Python OLS Regression model in Jupyter Notebook (6.2.0). By using the infection and no-infection categories as dependent variables, the variables that are statistically significant between the infection and no-infection groups were taken as independent variables for the model. Though the study showed significance with the p-value 0.05, the effect size is very low.
